# Parenting style, resilience, and mental health of community-dwelling elderly adults in China

**DOI:** 10.1186/s12877-016-0308-0

**Published:** 2016-07-08

**Authors:** Xue Zhong, Daxing Wu, Xueqing Nie, Jie Xia, Mulei Li, Feng Lei, Haikel A. Lim, Ee-Heok Kua, Rathi Mahendran

**Affiliations:** Medical Psychological Institute, The Second Xiangya Hospital of Central South University, 139 Middle Renmin Road, Changsha, Hunan 410011 People’s Republic of China; Department of Psychological Medicine, Yong Loo Lin School of Medicine, National University of Singapore, Singapore, Singapore

**Keywords:** Anxiety, Depression, Elderly, Parenting style, Resilience

## Abstract

**Background:**

Given the increasing elderly population worldwide, the identification of potential determinants of successful ageing is important. Many studies have shown that parenting style and mental resilience may influence mental health; however, little is known about the psychological mechanisms that underpin this relationship. The current study sought to explore the relationships among mental resilience, perceptions of parents’ parenting style, and depression and anxiety among community-dwelling elderly adults in China.

**Methods:**

In total, 439 community-dwelling elderly Chinese adults aged 60–91 years completed the Personal and Parents’ Parenting Style Scale, Connor–Davidson Resilience Scale, Zung Self-Rating Depression Scale, and Zung Self-Rating Anxiety Scale.

**Results:**

Elderly adults whose parents preferred positive and authoritative parenting styles had higher levels of mental resilience and lower levels of depression and anxiety. Elderly adults parented in the authoritarian style were found to have higher levels of depression and anxiety, with lower mental resilience.

**Conclusions:**

The findings of this study provide evidence related to successful ageing and coping with life pressures, and highlight the important effects of parenting on mental health. The results suggest that examination of the proximal determinants of successful ageing is not sufficient—distal factors may also contribute to the ‘success’ of ageing by modifying key psychological dispositions that promote adaptation to adversity.

**Electronic supplementary material:**

The online version of this article (doi:10.1186/s12877-016-0308-0) contains supplementary material, which is available to authorized users.

## Background

China has a rapidly ageing population: in 2010, one in 10, or more than 185 million, persons were aged ≥ 60 years; in 2050, this proportion will have increased to one in three [1]. In 2012, almost two in five elderly persons in China reported subclinical levels of depression [2]. Alleviating this potential burden may thus depend on uncovering the determinants of successful ageing [3]. Young and colleagues [4] described three domains of successful ageing: physiological (e.g. diseases and functional impairments), psychological (e.g. emotional vitality), and social (e.g. spirituality and adaptation through social support mechanisms). Ng and colleagues [5] defined successful ageing as good or excellent self-reported health status, independence in performing instrumental activities of daily life, Geriatric Depression Scale score ≤ 5, engagement in at least one social and one productive activity, and high reported level of life satisfaction. Previous research on successful ageing has focused heavily on biological and socio-demographic contributors to narrowly defined aspects of physical health [6], without giving much attention to the importance of psycho-social resources for mental and holistic well-being [5]. Mental resilience is a key psychosocial resource that has been shown to promote successful ageing [7].

Mental resilience is a positive personality characteristic that moderates the negative effects of stress and promotes adaptation, allowing individuals to thrive in the face of adversity [8]. It is enhanced by environmental factors, such as family and support systems [9]. Resilience is commonly perceived to be a good outcome despite adversity, or the ability to bounce back following adversity [10]. Windle [11] defined resilience as the process of effectively negotiating, adapting to, or managing significant sources of stress or trauma. Assets and resources of individuals, their lives, and the environment facilitate this capacity for adaptation, or ‘bouncing back’, in the face of adversity. Resilient individuals have lower levels of depressive symptomatology [12], and often feel that they have aged successfully [13]. Resilient elderly people often view their lives and health as satisfactory despite age-related disease and disability, and greater resilience, as assessed by the Connor–Davidson Resilience Scale (CD-RISC), has been related positively to key components of successful ageing [14]. These studies indicated that resilience may influence the development of human being in whole life.

Current models of mental resilience suggest that factors can be classified as internal (e.g. genetic) and external (e.g. environmental). Internal factors are generated from within an individual and include biological and psychological factors. External factors are extrinsic, and are reflected in the nature and quality of relationships established within and outside the family group [15]. For example, early experiences with parents have been shown to impact the well-being of elderly persons [16]. Based on parenting styles constructs developed by Baumrind [17–19], parenting styles are generally categorised along two axes (responsiveness to the child and demanding nature of the parent): authoritative parenting, where parents are demanding, but also responsive; authoritarian parenting, where parents are demanding and non-responsive; and permissive parenting, where parents are not demanding, but are extremely responsive. Contemporaneous research has brought to light the positive parenting style [20], in which parents focus on different strategies to create a positive environment based on mutual trust and respect. Study indicated that authoritative parenting has been shown to produce more successful adults in Western culture, with children experiencing authoritarian parenting showing more externalising behaviours and downstream psychiatric sequelae [21]. Among Chinese children, however, the authoritarian parenting style has been shown to produce the best outcomes (e.g. good school performance) [22, 23]. Regardless, greater parental care and lesser parental overprotection have been shown to contribute to increasing resilience, for example, in protecting adolescents from post-traumatic stress disorder symptoms [24]. Research has strongly suggested that such parenting styles are related to higher levels of mental resilience in children, even in late adolescence and young adulthood [15]. Zhang et al. [25] suggested that resilience has a significant negative predictive effect on depression in older adults. In addition, social support can enhance resilience in this population. Rothrauff et al. [26] conducted a telephoto-interview study to assess the associations of parenting behaviours remembered from childhood (classified as authoritative, authoritarian, indulgent, and uninvolved) with psychological well-being and depressive symptoms in mid- and later-life adults. They found that adults who remembered authoritative parents reported greater psychological well-being and fewer depressive symptoms than did those who remembered having authoritarian or uninvolved parents [26]. These means parenting style plays an important role in family relationships and it may influence individuals’ later development.

Previous study suggested that remembered parenting styles continue to be related to functioning across the lifespan [26] and resilience also influence human development and mental health [12, 13]. These indicated that parenting style and resilience may be correlated to mental health in whole life. However, little is known about the psychological mechanisms underpinning the relationships among these three factors. The purpose of our study was to examine the relationships among parenting style, mental resilience, and mental health in an elderly Asian population. We hypothesised that individuals with authoritarian parents would be less resilient and would report more depression and anxiety than would those whose parents used other styles and that mental resilience would mediate the relationship between perceived parents’ parenting style and mental health. The current study provides a unique perspective on the possible mediating role of mental resilience in the relationship between parents’ parenting style and mental health in community-dwelling elderly adults.

## Methods

### Participants and procedure

The study data were collected from a convenience sample of community-dwelling elderly adults in Hunan Province, China. The participants were mainly recruited from three senior activity centers (Wang Yue lake community, Ying Chun community and Xuan Feng community) located in different parts of Hunan province to increase the representativeness of the samples. We included only old adults aged ≥ 60 years who were able to understand and complete the questionnaire and provide voluntary consent. The old adults had raised offspring. Exclusion criteria were a diagnosis of mental illness and insufficient cognitive function for study participation (e.g. severe dementia). In consideration of potential literacy and visual limitations of elderly adults, the survey was conducted using the face-to-face interview method and a structured questionnaire. A sample of 439 (214 men, 225 women) elderly adults aged 60–91 (mean = 69.08, standard deviation = 7.25) years participated voluntarily in face-to-face interviews conducted by trained research assistants to assess parents’ parenting style, mental resilience, and anxious and depressive symptomatology. Table 1 provides demographic details of the study sample. All participants provided informed consent, and the Ethics Committee of the Second Xiangya Hospital of Central South University approved the study.

**Table 1:** Demographic characteristics of the study sample

	Total (*N* = 439)	Men (*n* = 214)	Women (*n* = 225)
Age, years (M ± SD)	69.08 ± 7.25	69.94 ± 7.35	68.26 ± 7.07
Education			
No formal schooling	40 (9.1)	5 (2.3)	35 (15.6)
Primary school	112 (25.5)	50 (23.4)	62 (27.6)
Middle school	120 (27.3)	56 (26.2)	64 (28.4)
High school	122 (27.8)	69 (32.2)	53 (23.6)
University	45 (10.3)	34 (15.9)	11 (4.9)
Current work status			
Retired	303 (69.0)	177 (82.7)	126 (56.0)
Casual labourer	15 (3.4)	13 (6.1)	2 (0.9)
Self-employed	13 (3.0)	11 (5.1)	2 (0.9)
Housewife	94 (21.4)	4 (1.9)	90 (40.0)
Full-time employee	14 (3.2)	9 (4.2)	5 (2.2)
Marital status			
Single or unmarried	3 (0.7)	1 (0.5)	2 (0.9)
Married	363 (82.7)	190 (88.8)	173 (76.9)
Divorced or separated	4 (0.9)	3 (1.4)	1 (0.4)
Widowed	69 (15.7)	20 (9.3)	49 (21.8)
Living arrangement			
Alone	26 (5.9)	9 (4.2)	17 (7.6)
With spouse	322 (73.3)	171 (79.9)	151 (67.1)
With son	60 (13.7)	23 (10.7)	37 (16.4)
With daughter	22 (5.0)	9 (4.2)	13 (5.8)
With grandchildren	3 (0.7)	1 (0.5)	2 (0.9)
With other relatives	2 (0.5)	1 (0.5)	1 (0.4)
With friends	2 (0.5)	1 (0.5)	2 (0.9)
In institution	2 (0.5)	0 (0)	2 (0.9)

Data are presented as *n* (%) unless otherwise indicated. M, mean; SD, standard deviation

### Measures

Parenting style was assessed using the Chinese version of the Personal and Parents’ Parenting Style Scale (PaPPS). This 13-item scale was developed in Singapore to examine the relationship between parents’ child-rearing strategies and how they were parented as children (see Appendix). It comprises four subscales (positive, authoritative, authoritarian, and permissive parenting styles), each of which has shown acceptable reliability (Cronbach’s alpha values) [27]. Respondents are asked to indicate the frequency with which their mothers and fathers engaged in specific behaviours, and the frequency with which they engaged in these behaviours toward their children. Responses are structured by a five-point scale ranging from never (1) to always (5). For the present study, mean subscale scores for both parents (when applicable) were used, with higher scores representing greater perceived frequency of a given parents’ parenting style. Cronbach’s alpha values for the subscales ranged from 0.78 to 0.90, indicating good reliability.

Mental resilience was measured with the Chinese version of the CD-RISC [28]. Responses to the instrument’s 25 items are structured by a five-point Likert-type scale (0–4). Examples of items are: ‘able to adapt to change’ and ‘tend to bounce back after illness or hardship’. Higher scores reflect greater resilience. The CD-RISC has demonstrated good internal consistency and test–retest reliability [9, 28]; the Cronbach’s alpha value for the present sample was 0.93.

Participants also completed the Chinese versions [29] of the Zung Self-Rating Depression Scale (SDS) [30] and the Zung Self-Rating Anxiety Scale (SAS) [31]. Responses to the 20 items of each scale are structured by a four-point Likert-type scale (1–4) that quantifies the levels of depressive and anxious symptomatology. Cronbach’s alpha values for both scales ranged from 0.83 to 0.84 in the present sample.

### Data analysis

Analyses were conducted with the SPSS software (version 19.0; SPSS Inc., Chicago, IL, USA) Pearson correlation coefficients were used to assess bivariate relationships. To determine mediation effects, we employed Wen and colleagues’ [32] procedure, as it best balances the sum of types 1 and 2 error rates and enables testing for partial and full mediation. To test our hypotheses, we controlled for confounding factors such as gender, age and living arrangement (e.g., alone, with spouse, with son, with daughter, with grandchildren, with other relatives, with friends, or in institution). First, the dependent variables (depression and anxiety) were regressed on the independent variables (four parenting styles); second, the hypothesised mediator (resilience) was regressed on the independent variables; and third, the dependent variable was regressed on the independent variables and hypothesised mediators in a single equation.

## Results

### Descriptive statistics and correlations of variables

*T*-tests showed no gender difference in participants’ or their parents’ parenting style (all *p* > 0.05). Significant gender differences were observed for depression (SDS score, *t* = 3.26, *p* < 0.01) and anxiety (SAS score, *t* = 3.37, *p* < 0.01); scores were higher among women than among men (Table 2). Table 3 presents the correlation matrix of the variables of interest. The positive parenting style was related to greater resilience (CD-RISC score, *r* = 0.36*, p* < 0.05), but not to anxiety or depression. The authoritative parenting style was related to greater resilience (*r* = 0.25, *p* < 0.01) and a lower level of depression (*r* = −0.10, *p* < 0.05), but not to anxiety. The authoritarian parenting style was related to lesser resilience (*r* = −0.17, *p* < 0.05), and higher levels of depression (*r* = 0.21, *p* < 0.01) and anxiety (*r* = 0.30, *p* < 0.01). The permissive parenting style was not related to resilience, anxiety, or depression. More resilient individuals were found have lower levels of depression (*r* = −0.31, *p* < 0.01) and anxiety (*r* = −0.23, *p* < 0.01).

**Table 2:** Descriptive statistics for depression, anxiety, and resilience in elderly adults

	Men	Women	*t*	*p*
Depression	45.78 ± 10.82	48.94 ± 10.15	−3.264	0.001
Anxiety	39.66 ± 9.67	42.90 ± 10.41	−3.374	0.001
Resilience	64.85 ± 13.77	62.33 ± 15.03	1.823	0.069

Values are expressed as mean ± standard deviation

**Table 3:** Correlations between PaPPS subscale, CD-RISC, SDS, and SAS scores

	1	2	3	4	5	6
1. Positive parenting style	1					
2. Authoritative parenting style	0.752**	1				
3. Authoritarian parenting style	−0.073	−0.029	1			
4. Permissive parenting style	0.102*	0.290**	0.029	1		
5. Resilience	0.360**	0.245**	−0.173**	0.007	1	
6. Depression	−0.077	−0.095*	0.212**	−0.086	−0.309**	1
7. Anxiety	−0.020	−0.021	0.296**	−0.051	−0.229**	0.623**

*p < 0.05, **p < 0.01

### Mediating effects

**Fig. 1:**
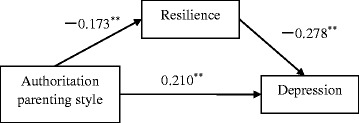
Path model of the relationships among authoritarian parenting style, resilience, and depression. Values presented are standardised regression coefficients

**Fig. 2:**
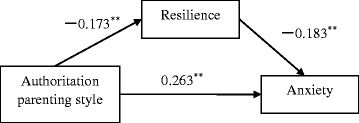
Path model of the relationships among authoritarian parenting style, resilience, and anxiety. Values presented are standardised regression coefficients

The hierarchical regression analysis showed that the authoritarian parenting style was the only significant predictor of both depression and anxiety (*β*_*SDS*_ = 0.21, *β*_*SAS*_ = 0.29, *p* < 0.001), and a significant predictor of low resilience (*β* = −0.12, *p <* 0.001; Table 4). Low mental resilience predicted depression and anxiety, and partially mediated the relationships between authoritarian parenting style and depression (16 % of the total effect) and anxiety (7.36 % of the total effect). Figs. 1 and 2 illustrate the path model of the relationships among authoritarian parenting style, resilience, and depression/anxiety.

**Table 4:** Mediating effects of resilience on relationships with authoritarian parenting style

Measure	Step	Standardised regression equation	SE	*t*
Depression	1	*y* = 0.210*x*	0.266	4.499**
	2	*m* = 0.173*x*	0.366	−3.681**
	3	*y* = −0.278 *m*	0.033	−6.087**
		+0.162*x*	0.070	3.479**
Anxiety	1	*y* = 0.295*x*	0.250	6.454**
	2	*m* = −0.173*x*	0.366	−3.681**
	3	*y* = −0.183 *m*	0.032	−4.006**
		+0.263*x*	0.250	5.770**

**p < 0.01

*SE*, standard error of the mean

In depression: mediating effect of resilience between authoritarian parenting style and depression

y: authoritarian parenting style; m: resilience; x: depression

Step 1: The dependent variable (depression) was regressed on the independent variables (authoritarian parenting style)

Step 2: the hypothesized mediator (resilience) was regressed on the independent variables (authoritarian parenting style)

Step 3: the dependent variable (depression) was regressed on both the independent variable (authoritarian parenting style) and mediators (resilience) in one equation

In anxiety: mediating effect of resilience between authoritarian parenting style and anxiety

y: authoritarian parenting style; m: resilience; x: anxiety

Step 1: The dependent variable (anxiety) was regressed on the independent variables (authoritarian parenting style)

Step 2: the hypothesized mediator (resilience) was regressed on the independent variables (authoritarian parenting style)

Step 3: the dependent variable (anxiety) was regressed on both the independent variable (authoritarian parenting style) and mediators (resilience) in one equation

## Discussion

The results of the present study demonstrate that mental resilience may mediate the relationships between certain parenting styles and mental health in later life in a sample of community-dwelling elderly Chinese adults. The family setting is the initial context for individuals’ development, and parenting styles may mould mental resilience. The authoritative parenting style, which involves warmth and responsiveness, has been related consistently to positive developmental outcomes, including fewer behavioural problems and psychological symptoms. Similar to studies conducted in other populations [21], our results suggest that warm, supportive, and loving parenting, encapsulated by the positive and authoritative parenting styles, is associated with the development of mental resilience, and perhaps even the maintenance of resilience in later life [15]. The authoritarian parenting style, in contrast, involves a low degree of responsiveness and high level of demandingness. Authoritarian parents are often cold, unsupportive, insensitive to their children’s needs, and demanding in their control. In our study, this parenting style was related to less resilience and more depression and anxiety in comparison with other parenting styles. Although other studies conducted with Chinese samples have shown that the authoritarian parenting style results in better mental health outcomes [22, 23], they involved first- and second-generation immigrants to America, and may not be representative of the cultural norms in China.

Our results also showed that participants who reported more depressive and anxiety symptoms has less mental resilience, in line with findings from other contemporary studies [12, 13]. Mental resilience may thus be regarded as a protective factor that may increase the ability to overcome negative life events and crises, and increase individuals’ willingness to seek mental health care [33, 34]. Resilience increases the likelihood that a person will talk with health professionals about depressive symptoms and seek care to relieve those symptoms. Resilience can impact health and well-being and is an important aspect of older individuals’ physical and psychological adjustment and adaptation to the ageing process [11, 35]. The present findings thus also contribute to the growing literature recognising the importance of mental resilience in improving well-being in later years [14].

In line with our hypotheses, our results suggest that mental resilience mediates the relationships between some parenting styles and anxious and depressive symptomatology. The lack of warmth in the authoritarian parenting style may result in low mental resilience and subsequent psychiatric sequelae. Consistent with previous studies, we found that mental resilience as an important protective psychological resource shaped by the style of the parent–child relationship. Acceptance–involvement (positive and authoritative) parenting styles have been found to be positive predictors of mental resilience [15], whereas the authoritarian style has frequently been associated with low resilience [36]. Social cohesion, belonging, and changes therein were found to predict the social and physical well-being of community-dwelling older people in the Netherlands over time [37]. Collaborations between health care professionals and community workers in the health and social sectors would extend community outreach and support [38]. Stronger ties among families and a sense of belonging are thus needed.

Finally, the results of this study suggest that examination of the proximal determinants of successful ageing is not sufficient—distal determinants, such as how individuals were parented, seem to contribute on some level to the ‘success’ of older adults’ ageing by modifying key psychological dispositions that promote adaptation to adversity. Recognition of the limitations of this study is, however, important. Although participants had completed their ‘parental duties’ and constitute a significant proportion of the population of interest (elderly adults), their responses about their parents’ parenting style may be subject to recall bias or romanticisation based on their own parenting experiences. These practical limitations, however, do not detract from the study findings, which may generate hypotheses for future research. In addition, the sample was relatively small. Moreover, differences among research assistants conducting interviews, in terms of personality and language used, may have served as confounding factors that influenced participants’ responses. Finally, the cross-sectional survey used in the study did not enable examination of the causality of the effects of psychosocial factors on geriatric depression. Thus, longitudinal studies would help to clarify the predictive effects of these risk factors on late-life mental health.

## Conclusion

The findings of the present study suggest that resilience mediates the relationship between parenting style and mental health in community-dwelling elderly Chinese adults. As expected, adults who remembered more authoritarian parenting reported lower resilience, and more depressive and anxious symptoms. Resilience is a coping style applied in relation to stress and depression, and it plays an important role in human development. Parenting styles continue to be related to functionality throughout the life span. The findings of this study provide additional evidence highlighting the important effects of parenting on mental health.

## Abbreviations

CD-RISC, Connor–Davidson Resilience Scale; PaPPS, Personal and Parents’ Parenting Style Scale; SAS, Zung Self-Rating Anxiety Scale; SDS, Zung Self-Rating Depression Scale

## Appendix

**Personal and Parents’ Parenting Style (PaPPS)**

**English version of Singapore**

Please rate the different parenting practices listed below. Scores range from “Never” to “Always” on a 5-point scale. At the end of each section, add up the scores and divide it by the number of questions in that section.

F = my Father, M = my Mother, I = myself (if you’re a parent).

A. **Positive Parenting Style**
  1. My parents encouraged me in my career. (I encourage my kids in their career.)
  2. My parents talked to me about values. (I talk to my kids about values.)
  3. My parents talked to me about the family history.(I talk to my kids about the family history.)
B. **Authoritative Parenting Style**
  1. My parents were responsive to my feelings and needs. (I am responsive to my kids’ feelings and needs.)
  2. My parents encouraged me to talk about my feelings and problems. (I encourage my kids to talk about their feelings and problems.)
  3. My parents complimented me.(I compliment my kids.)
C. **Authoritarian Parenting Style**
  1. My parents shouted when he/she disapproved of my behaviour. (I shout when I disapprove of my kids’ behaviour.)
  2. My parents spanked me when I didn’t like what he/she did or said. (I spank my kids when they don’t like what I do or say.)
  3. My parents openly criticized me when my behaviour did not meet his/her expectations. (I openly criticize my kids when their behaviour does not meet my expectations.)
D. **Permissive Parenting Style**
  1. My parents gave into me when I caused a commotion. (I give into my kids when they cause a commotion.)
  2. My parents spoilt me. (I spoil my kids.)
  3. My parents ignored my bad behaviour. (I ignore my kids’ bad behaviour.)
E. **Overall Parenting Assessment**
  - Looking back, I am happy with my parents.

## Additional file

Additional file 1: Table S1. Datasets. (XLSX 121 kb)
